# Methods in molecular biology: minor errors in primer citations with major consequences: how can we minimize these mistakes? – reply

**DOI:** 10.1054/bjoc.1999.1187

**Published:** 2000-04-03

**Authors:** D R Edwards

**Affiliations:** School of Biological Sciences, University of East Anglia, Norwich, Norfolk NR4 7TJ, UK


We were interested in the characteristics of Forsyth's primer pair and checked it by using the software 'Primer 3' as found on the internet (http://www.genome.wi.mit.edu/cgi-bin/primer/primer3_www.cgi). 'Primer 3' picks polymerase chain reaction (PCR) primers amplifying a particular region of a target gene defined from a nucleotide sequence database, generates scores for complementarities of the primer pairs and calculates melting temperatures and GC-contents of primers and products. Important for that is the complete entry of mRNA sequence of the target gene from a molecular biology database (Burks, 1999).

In the case of the MMP-2 primers from Forsyth et al, 'Primer 3' could not identify the binding sites of both primers in the source MMP-2 mRNA sequence (Accession number [AC]: JO3210; identification code [ID]: HUMCN4GEL). Putative binding sites of both MMP-2 primers were therefore checked by a Blast-Internet program (http://www.ncbi.nlm.nih.gov/cgi-bin/BLAST/nph-new-blast) comparing a nucleotide query sequence against a nucleotide sequence database. The applied software falls back on four databases (non-redundant sequences from GenBank, EMBL, DDBJ and PDJ). To our surprise, the used MMP-2 primer sequences of Forsyth et al produced significant alignments with regions of the human tissue inhibitor TIMP-1 gene, exon 1 (AC: L47357; ID: HUMTIMP1G), the human collagenease inhibitor mRNA (AC: M59906; ID: HUMOGCA) and the human fibroblast collagenase inhibitor mRNA (AC: M12670; ID: HUMFCI) but not with the MMP-2 gene. However, the alignments with both collagenase inhibitors were found only in 22 of the 25 input bases of the primers and the Blast-program automatically cut off the first three bases.

After comparing the source mRNA sequence for both collagenase inhibitors mentioned above and the completed sequences of the primers, we found one false base at the third position of the 5′- (A instead of G) and at the 3′-primer (C instead of A) both at the 5′-end. We also observed a print error in the reading direction of the gelatinase A reverse primer (5′ instead of 3′). Consequently, the size of the amplification product would be 686 bp instead of the published product size of 473 bp.

In consequence of our investigation, we are uncertain whether the authors really detected MMP-2 and not TIMP-1. The agarose gel bands shown in Figure 1 cannot be considered as proof for the detection of the target gene and for the correct PCR product size since no DNA molecular weight markers are present in the same gel run.

In addition to these data, we found that the product size for MT1-MMP as cited in the same paper is not 548 bp but 530 bp and that the used 5′-primer for this gene contains a false cited base in the third position from the 3′-end (G instead of C).

Apart from drawing the interested reader's attention to these possible mistakes, the paper gives rise to the following general recommendations:

- The selection of primers has to be documented in an easily understandable manner to avoid serious mistakes
- Therefore, primer sequences should be specified by declaration of the source of the target gene sequence (e.g. the nucleotide sequence database used) and of the primer position within the target sequence
- In addition, citation of the accession number (AC) and the identification code (ID) of the investigated gene sequence would give a definite relation between a nucleotid sequence and its target gene.

Printing errors of nucleotide sequences of primers could thus more easily be checked, and the suggested procedure gives a better guarantee for correct citations and successful application of published methods in molecular biology.

*M Jung[1], JM Muche[2], K Jung[1] and SA Loening[1]*
*Departments of [1]Urology and [2]Dermatology,*
*University Hospital Charité,*
*Humboldt University Berlin, Schumannstr. 20/21,*
*D-10098 Berlin, Germany*

#### REFERENCES

Burks C (1999) Molecular Biology Database List. *Nucleic Acids Res* **27**: 1–9
Forsyth PA, Wong H, Laing TD, Rewcastle NB, Morris DG, Muzik H, Leco KJ, Johnston RN, Brasher PMA, Sutherland G and Edwards DR (1999) Gelatinase-A (MMP-2), gelatinase-B (MMP-9) and membrane type matrix metalloproteinase-1 (MT1-MMP) are involved in different aspects of the pathophysiology of malignant gliomas. *Br J Cancer* **79**: 1828–1835
Parsons SL, Watson SA, Brown PD, Collins HM and Steele RJC (1997) Matrix metalloproteinases. *Br J Surg* **84**: 160–166
Wittwer CT, Ririe KM, Andrew RV, David DA, Gundry RA, and Balis UJ (1997) The LightCycler™: a microvolume multisample fluorimeter with rapid temperature control. *Bio Techniques* **22**: 176–181

DOI: 10.1054/ bjoc.1999.1187, available online at http://www.idealibrary.com on IDEAL®

### Methods in molecular biology: minor errors in primer citations with major consequences: how can we minimize these mistakes? – reply

**Sir**,
I am replying to the above letter, which was sent concerning our paper 'Gelatinase-A (MMP-2), gelatinase-B (MMP-9) and membrane type-matrix metalloproteinase-1 (MT1-MMP) are involved in different aspects of the pathophysiology of malignant gliomas', Forsyth PA et al (1999) *Br J Cancer* **79**: 1828–1835.

Jung et al point out that the primer sequences listed in our paper for reverse transcription polymerase chain reaction (RT-PCR) amplification of human gelatinase-A (MMP-2) are incorrect. The sequences given in fact correspond to primer sequences used for human TIMP-1 amplification, with additional *Bam*HI and *Hin*DIII cloning sites at the 5′-end of the oligonucleotides to allow

 *© 2000 Cancer Research Campaign*

sub-cloning. This arose due to a mistake in transcribing information from a larger table of MMP/TIMP oligonucleotide primer sequences when the *British Journal of Cancer* paper was being put together. However, this does not compromise the data reported in the paper, which were obtained using the correct MMP-2 primer sequences forward = 5′-GGCCCTGTCACTCCTGAGAT, reverse = 5′-GGCATCCAGGTTATCGGGGA, which as described amplify a 474 bp PCR product. The methods that we have used for MMP and TIMP quantification by RT-PCR are described in detail in Wong et al (in press).

We are grateful to Jung et al for pointing out this error, and the correct size of the MT1-MMP PCR product as 530 bp. There is certainly a need for vigilance in the use of PCR as a research tool, which we maintain in our own laboratories by subcloning and sequence analysis of RT-PCR products to confirm their identities. We agree that it is useful to identify the target gene sequences used and the positions of primer pair combinations within those sequences as a method of facilitating studies by other labs. Consequently we have drawn up this information for the MMP-2, MMP-9 and MT1-MMP targets used in our paper (Table 1). However, we caution that even with such information in hand, it is still necessary for other laboratories to confirm independently the identities of the PCR products that they obtain, using appropriate molecular criteria.

*DR Edwards*
*School of Biological Sciences, University of East Anglia, Norwich, Norfolk NR4 7TJ, UK*

**REFERENCES**

Forsyth PA, Wong H, Laing TD, Rewcastle NB, Morris DG, Muzik H, Leco KJ, Johnston RN, Brasher PMA, Sutherland G and Edwards DR (1999) Gelatinase-A (MMP-2), gelatinase-B (MMP-9) and membrane type matrix metalloproteinase-1 (MTI-MMP) are involved in different aspects of the pathophysiology of malignant gliomas. *Br J Cancer* **79**: 1828–1835

Wong H, Muzik H, Groft LL, Lafleur MA, Matouk C, Forsyth PA, Schultz GA, Wall SJ and Edwards DR (2000) Monitoring MMP and TIMP mRNA expression by RT-PCR. In: *Matrix metalloproteinase protocols*, Clark IM, (ed). Humana Press, Totoya, NJ

**Table 1** RT-PCR primer description

| Target gene | Primer sequence | Target accession number | Position | Product size (bp) | PCR cycle no. |
|---|---|---|---|---|---|
| Gelatinase-A | GGCCCTGTCACTCCTGAGAT | J03210 | 1337–1356 | 474 | 29 |
| (MMP-2) | GGCATCCAGGTTATCGGGGA | | 1810–1791 | | |
| Gelatinase-B | TGGACGATGCCTGCAACGTG | NM 004994.1 | 1554–1573 | 455 | 33 |
| (MMP-9) | GTCGTGCGTGTCCAAAGGCA | | 2008–1989 | | |
| MT1-MMP | GCCCATTGGCCAGTTCTGGCGGG | NM 004995.1 | 1178–1200 | 530 | 30 |
| (MMP-14) | CCTCGTCCACCTCAATGATGATC | | 1707–1685 | | |
| GAPDH | CGGAGTCAACGGATTTGGTCGTAT | M33197 | 78–101 | 307 | 23 |
| | AGCCTTCTCCATGGTGGTGAAGAC | | 384–361 | | |

DOI: 10.1054/ bjoc.1999.1188, available online at http://www.idealibrary.com on IDEAL®

### Prognostic value of histological and biological markers in pharyngeal squamous cell carcinoma: a case-control study

**Sir**,
We have read with great interest the article of Guerry and co-workers ‘Prognostic value of histological and biological markers in pharyngeal squamous cell carcinoma: a case-control study’ (Guerry et al, 1998). The authors present a comparison between some histological features (grading, keratinization and vascular emboli) and immunohistochemical features (expression of p53, c-*erb*-B2, Rb and bcl-2) of primary tumour biopsies in two groups of patients affected by pharyngeal cancer: patients who developed distant metastasis (DM) and patients who did not. In the case-control design each patient who developed a DM was matched to a control patient with the same tumour site, the same nodal size and level in the neck, and with an equal follow-up but free of DM. Out of 65 patients there were 45 with positive neck nodes. It was found that the risk for DM was halved in patients with tumours expressing c-*erb*-B2 compared with patients with tumours negative for c-*erb*-B2.

This result is of particular interest because it gives new perspective in the treatment planning and prognosis for these patients. However, the design of this case-control study is not completely correct.

Indeed, the two groups of patients with and without DM are only clinically homogeneous. No data regarding the immunohistochemical homogeneity of the neck nodes was presented. This is

© 2000 Cancer Research Campaign

